# Prognostic Value of CT Imaging-Based Tumor Volume in Patients With Non-Surgical Esophageal Squamous Cell Carcinoma

**DOI:** 10.3389/fonc.2020.602681

**Published:** 2021-01-22

**Authors:** Ning Kang, Yeying Fang, Huijun Zhu, Zhiling Shi, Liuyin Chen, YuShuang Lu, Housheng Wang, Jiamei Lu, Wenqi Liu, Kai Hu

**Affiliations:** ^1^ Department of Radiation Oncology, The First Affiliated Hospital of Guangxi Medical University, Nanning, China; ^2^ Department of Radiation Oncology, The Second Affiliated Hospital of Guangxi Medical University, Nanning, China; ^3^ Department of Oncology, Yue Bei People’s Hospital, Shaoguan, China; ^4^ Graduate School of Guangxi Medical University, Nanning, China

**Keywords:** tumor volume, CT imaging, non-surgical, esophageal squamous cell carcinoma, prognosis

## Abstract

**Background:**

The American Joint Committee on Cancer-Tumor (AJCC-T) staging system for esophageal carcinoma patients, which is based on the depth of tumor invasion, is not applicable in some cases. This study aims to assess the prognostic value of CT imaging-based tumor volume and its usefulness for T staging in patients with non-surgical esophageal squamous cell carcinoma (ESCC).

**Methods:**

We retrospectively reviewed the records of 158 ESCC patients undergoing definitive (chemo) radiotherapy from two hospitals. Tumor volume based on the CT imaging was calculated using the formula: V = π*abc* / 6. Three cutoff points for tumor volume were obtained with the X-tile software. Overall survival (OS) was analyzed using the Kaplan–Meier method. The -2 log-likelihood ratio and Akaike Information Criterion (AIC) value were evaluated to compare the AJCC-T staging system with the proposed T staging method.

**Results:**

The median tumor volume was 19.8 cm³ (range from 1.0 to 319.5 cm³). The three optimal cutoff points of tumor volume were 12.7, 22.8, and 51.9 cm³, and the patients were divided into four groups named as proposed T1–T4 stages. The 3-year OS rates in patients with proposed T1 to T4 stages were 67.9%, 30.6%, 21.3%, and 5.3%, respectively. The −2 log-likelihood ratios of the AJCC-T stage and proposed T stage were 1,068.060 and 1,047.418, respectively. The difference in the AIC value between the two T staging systems was 18.642.

**Conclusion:**

CT imaging-based tumor volume was superior to the depth of tumor invasion for T staging in predicting the prognosis of non-surgical ESCC patient.

## Introduction

According to the latest global cancer incidence and mortality statistics, it was estimated that there would be 572,034 new cases of esophageal carcinoma (EC) diagnosed in 2018 worldwide, resulting in an estimated 508,585 related deaths (1). It was also reported that there were about 477,900 new EC cases and 375,000 deaths caused by this cancer in China in 2015, and it had the third highest incidence rate and the fourth highest mortality rate (2). Thus, EC is regarded as a severe public health concern both in China and worldwide. In addition, owing to the lack of typical symptoms and low screening rates, 40–60% of the patients with EC were diagnosed with an advanced stage and were not suitable for radical surgery (3).

In the current AJCC-tumor-node-metastasis (TNM) staging system, the T stages of EC patients are defined as the depth of tumor invasion. However, some EC patients are unresectable so that the depth of tumor invasion is occasionally difficult to determine accurately. Endoscopic ultrasonography (EUS) is the best method to determine the extent of local early lesions, but this method fails sometimes because luminal stenosis is not rare in EC patients (4). Moreover, some studies have shown that the preoperative EUS stage was not consistent with the postoperative pathological stage (5, 6). On the other hand, unlike in Western countries, where esophageal adenocarcinoma (EAC) is predominant, approximately 90% of EC is squamous cell carcinoma in China (7, 8). Consequently, the current staging system which is mainly based on the survival data of EAC may not be applicable for esophageal squamous cell carcinoma (ESCC) (9). Given these factors, it is necessary to develop an accurate and feasible staging system for patients with non-surgical ESCC.

This study aims to develop a simple and applicable T staging method for patients with non-surgical ESCC that can represent their prognosis better than the AJCC-T staging system. To this end, the tumor volume was calculated by retrospectively analyzing its dimensions on CT imaging before treatment, and the appropriate cutoff point was determined using statistical software in the present study. Then, the proposed T stages were redefined according to the tumor volume and were compared with the AJCC-T stages to access the rationality of using CT imaging-based tumor volume as T stages.

## Materials and Methods

### Patients

A total of 158 patients with non-surgical ESCC treated with definitive (chemo) radiotherapy in the radiation oncology department of the First and the Second Affiliated Hospital of Guangxi Medical University between January 2010 and December 2015 were enrolled. All patients were recruited based on the following selection criteria: (i) diagnosed with pathologically confirmed ESCC; (ii) aged ≥18 years and ≤80 years; (iii) underwent definitive (chemo) radiotherapy initially and without any prior treatments; (iv) had complete information for staging; (v) had no other malignancy or distant metastasis (M0); (vi) had completed the treatment plan; (vii) had no severe cardiopulmonary insufficiency; and (viii) had an Eastern Cooperative Oncology Group performance status of 0–2. This study was approved by the Medical Ethics Committee of the hospitals. The requirement for written informed consent was waived owing to the retrospective nature of this study.

### Clinical Staging

After the pathological determination of ESCC, staging modalities included barium esophagography, upper gastrointestinal endoscopy, EUS, and CT scans of the cervix, thorax, and abdomen using intravenous contrast. Bronchoscopy, ^18^F-fluorodeoxyglucose positron emission tomography-CT, and EUS-guided fine-needle aspiration were used for a proportion of the patients.

The criteria for lesions were the localized or circumferential thickening of the esophageal wall, the thickness of the esophageal wall exceeding 5 mm, the diameter of the non-gasless esophagus exceeding 10 mm, or irregular local lumen stenosis. The location and length of the lesions, as determined by esophageal barium meal examination, were also considered. The lesion length (*a*) of tumor invasion in the superoinferior direction and the longest (*b*) and the shortest (*c*) diameters at the maximum transverse plane of the tumor were comprehensively evaluated, and then the tumor volume was calculated using the formula: V = π*abc* / 6 (10, 11). Lesion measurements were performed independently by two radiation oncologists (deputy chief physician or above) on CT imaging. A third reviewer was consulted when there was a volume variation of more than 10%. In order to restage the patients accurately according to the tumor volume, the clinical information of the patients was collated into a database.

The TNM classification was first performed according to the 6^th^ edition AJCC staging system for ESCC. The X-tile software (version 3.6.1, Yale University School of Medicine, New Haven, CT, USA) was applied to obtain three appropriate cutoff points for tumor volume, and then the patients were divided into four groups named as proposed T1–T4 stages. Subsequently, we used the proposed T stages instead of the T stages in the 6^th^ edition AJCC-TNM clinical staging system to determine the modified clinical stages.

### Treatment Protocols

External beam radiation was delivered using either three-dimensional conformal radiotherapy technique or intensity-modulated radiation therapy for all patients. The gross tumor volume (GTV) of the esophagus was defined according to the aforementioned imaging examinations. The involved lymph nodes (GTVnd) were defined as lymph nodes with a short diameter of ≥5 mm for paraesophageal, tracheoesophageal groove, and pericardial angle lymph nodes, while for other metastatic lymph nodes it was a short diameter ≥10 mm. The clinical target volume (CTV) was generated with a 2–4 cm margin in the superoinferior direction and 5–10 mm margins were left in the anteroposterior and lateral directions around the primary GTV.

For cervical and upper thoracic tumors, the CTV included supraclavicular and paraesophageal lymph nodes, and those in one, two, four, and seven lymph node stations. For middle thoracic tumors, the CTV included tracheoesophageal sulcus, paraesophageal lymph nodes, and those in one, two, four, seven, and eight lymph node stations. For lower thoracic tumors, the CTV included lymph nodes located at stations four, seven, and eight and paraesophageal, perigastric, and celiac axis regions.

The planning target volume (PTV) was generated by adding a 5 mm margin to the target. The radiation dose delivered to 95% PGTV was 60–64 Gy (2.0–2.2 Gy per fraction) and 50.4–54 Gy (1.8–2.0 Gy per fraction) to 95% PCTV in 28–32 fractions. The radiation was delivered five times a week.

All patients were recommended to receive concurrent and adjuvant platinum-based chemotherapy (plus paclitaxel or 5-fluorouracil) every three weeks. Sixty-two patients refused chemotherapy, whereas the remaining 96 patients received at least one cycle of chemotherapy.

### Follow-Up

All patients were followed up every three months in the first two years and every six months until five years, then annually thereafter. Regular follow-up regimens included physical, laboratory, imaging, and endoscopic examinations for assessing recurrence or metastasis. Overall survival was defined as the time between the date of the beginning of treatment and the date of death or the last follow-up. The patients were mainly followed up *via* telephone, and the outpatient and inpatient records were reviewed. The follow-up period was up to August 1, 2018.

### Statistical Analysis

The optimal cutoff point of tumor volume was obtained using the X-tile software. Overall survival rates in univariate analysis were calculated using the Kaplan–Meier method. Survival curves were compared using the log-rank test. Multivariate analyses were performed using Cox regression models, and p ≤0.05 was considered statistically significant. All statistical analyses were performed using SPSS software (version 23.0, SPSS Inc., Chicago, IL, USA).

## Results

### Patient Characteristics

Among the 158 patients recruited in this study, 140 (88.6%) were men, and 18 (11.4%) were women. The median age of the patients was 58 years (range from 39 to 80 years). The demographic and clinicopathological characteristics of the patients are summarized in **Table 1**.

**Table 1:** Clinicopathological Characteristics of patients and Results of Univariate Analysis for 3-year OS.

Variables	Number of patients (%)	3-Year Survival (%)	Total P Value	HR (95%CI)	P Value
Age			0.581		
<60	81(51.3%)	30.6%		1 (reference)	–
≥60	77(48.7%)	25.9%		1.102(0.759–1.601)	0.582
Sex			0.121		
Male	140 (88.6%)	35.6%		1 (reference)	–
Female	18 (11.4%)	55.6%		1.623(0.870–3.029)	0.122
Tumor location			0.913		
Cervical	7(4.4%)	42.9%		1 (reference)	–
Upper	44(27.8%)	39.1%		1.247(0.285–2.643)	0.678
Middle	87(55.1%)	39.0%		1.331(0.484–3.662)	0.580
Lower	20(12.7%)	39.8%		1.151(0.378–3.503)	0.804
6^th^ T stage			0.049		
T1	3(1.9%)	66.7%		0.309(0.043–2.239)	0.245
T2	30 (19.0%)	54.1%		0.485(0.277–0.849)	0.011
T3	66(41.8%)	36.6%		0.837(0.556-1.260)	0.395
T4	59(37.3%)	34.0%		1 (reference)	–
6^th^ N stage			<0.001		
N0	60(38.0%)	74.9%		1 (reference)	–
N1	98(62.0%)	17.5%		4.434(2.835–6.936)	0.000
6^th^ Clinical stage			<0.001		
I	2(1.2%)	100%		–	0.963
II	33(20.9%)	60.2%		0.291(0.172–0.493)	0.000
III	57(36.1%)	51.8%		0.299(0.193–0.662)	0.000
IV	66(41.8%)	16.5%		1 (reference)	–
Proposed T stage			<0.001		
T1	57(36.1%)	67.9%		0.107(0.058–0.197)	0.000
T2	35(22.1%)	30.6%		0.235(0.128–0.434)	0.000
T3	47(29.8%)	21.3%		0.412(0.236–0.718)	0.002
T4	19(12.0%)	5.3%		1 (reference)	–
Modified Clinical stage			<0.001		
I	31(19.6%)	83.9%		0.138(0.075–0.254)	0.000
II	41(25.9%)	45.3%		0.273(0.166–0.488)	0.000
III	37(23.4%)	25.6%		0.366(0.225–0.595)	0.000
IV	49(31.1%)	16.7%		1 (reference)	–
Tumor length (a)			0.000		
<7cm	75(47.5%)	52.8%		1 (reference)	–
≥7cm	83(52.5%)	25.6%		2.005(1.371–2.933)	0.000
Max diameter (b)^*^			0.000		
<3cm	66(41.8%)	59.1%		1 (reference)	–
≥3cm	92(53.2%)	22.7%		2.561(1.716–3.823)	0.000
Min diameter (c)^#^			0.000		
<1.8cm	74(46.8%)	61.9%		1 (reference)	–
≥1.8cm	84(53.2%)	18.8%		3.252(2.188–4.834)	0.000
Chemotherapy			<0.001		
YES	96 (60.8%)	49.7%		1 (reference)	–
NO	62 (39.2%)	22.4%		2.374(1.627–3.466)	0.000

*The longest diameter on the maximum transverse plane of the tumor.

^#^The shortest diameter on the maximum transverse plane of the tumor.

The median tumor volume was 19.8 cm³ (range from 1.0 to 319.5 cm³) as calculated using the formula: V = π*abc* / 6. The three optimal cutoff points of tumor volume were 12.7, 22.8, and 51.9 cm³ using the X-tile software. The patients were divided into four groups defined as follows: 57 cases of T1 (tumor volume <12.7 cm³), 35 cases of T2 (12.7cm³ ≤tumor volume <22.8 cm³), 47 cases of T3 (22.8 cm³ ≤tumor volume <51.9 cm³), and 19 cases of T4 (tumor volume ≥51.9 cm³). One hundred and sixteen patients were changed from the AJCC-T stage to proposed T stage as summed up in **Table 2**. This led to 85 patients being migrated between the 6^th^ AJCC TNM staging system and the modified staging method as summed up in **Table 3**.

**Table 2:** Cross table of T stage for patients according to the 6^th^ AJCC-T staging system and the proposed T staging method.

T stage	Proposed T1	Proposed T2	Proposed T3	Proposed T4	Total
6^th^ AJCC T1	2	0	0	1	3
6^th^ AJCC T2	20	3	5	2	30
6^th^ AJCC T3	24	15	24	3	66
6^th^ AJCC T4	11	17	18	13	59
Total	57	35	47	19	158

**Table 3:** Cross table of clinical stage for patients according to the 6^th^ AJCC-TNM staging system and the modified staging method.

Clinical stage	Modified I	Modified II	Modified III	Modified IV	Total
6^th^ AJCC I	2	0	0	0	2
6^th^ AJCC II	10	16	5	2	33
6^th^ AJCC III	15	14	18	10	57
6^th^ AJCC IV	4	11	14	37	66
Total	31	41	37	49	158

In addition, the tumor length (a) ranged from 1.6 to 18.5 cm (mean, 7.2 cm; median, 7.0 cm), and the longest (b) and the shortest (c) diameters on the maximum transverse plane of the tumor ranged from 1.2 to 8.0 cm (mean, 3.2 cm; median, 3.0 cm) and 0.7 to 5.4 cm (mean, 1.9 cm; median, 1.8 cm), respectively (**Table 4**).

**Table 4:** Characteristics of the tumor.

Characteristic	Mean ± SD	Range
Tumor length (a) (cm)	7.2 ± 2.8	1.6–18.5
Max diameter (b)^*^ (cm)	3.2 ± 1.2	1.2–8.0
Min diameter (c)^#^ (cm)	1.9 ± 0.8	0.7–5.4
Tumor volume (cm^3^)	30.2 ± 39.8	1.0–319.5

^*^ The longest diameter on the maximum transverse plane of the tumor

^#^ The shortest diameter on the maximum transverse plane of the tumor

### Overall Survival Rates

During the period of analysis, 111 patients (70.3%) died. The median follow-up time was 29 months (range from 3.0 to 93.0 months), and the 3- and 5-year OS rates were 39.0 and 17.0%, respectively.

**Figure 1:**
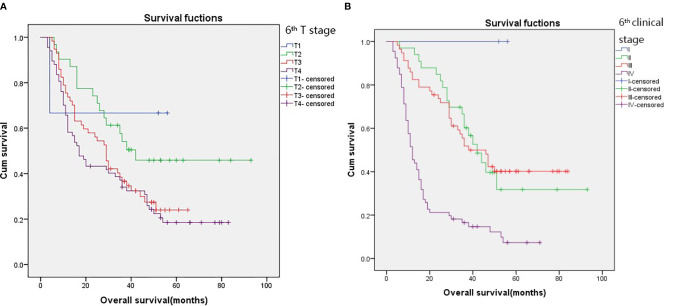
Kaplan–Meier survival curves for patients stratified on basis of the 6^th^ T stages **(A)** and 6^th^ clinical stage **(B)**.

The 3-year survival rates in patients with T1, T2, T3, and T4 stages according to the 6^th^ edition AJCC-T staging system were 66.7%, 54.1%, 36.6%, and 34.0%, respectively (**Figure 1A**; p = 0.049). However, there were no significant differences in survival between patients in T3 and T4 subgroups. Overlapping survival curves were also observed between patients with stage II and III disease (**Figure 1B**; p < 0.001).

**Figure 2:**
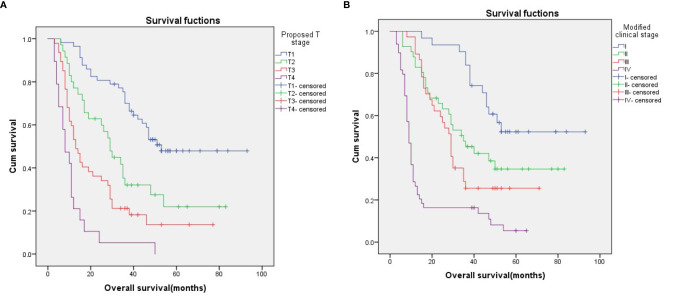
Kaplan–Meier survival curves for patients stratified on basis of the proposed T stages **(A)** and the modified clinical stage **(B)**.

Nevertheless, Kaplan–Meier survival curve analysis based on the proposed T stage and modified clinical stage indicated that they had good discriminatory ability in each subgroup, as they showed a relatively distinct distribution of survival (**Figures 2A, B**; p < 0.001 for both).

**Figure 3:**
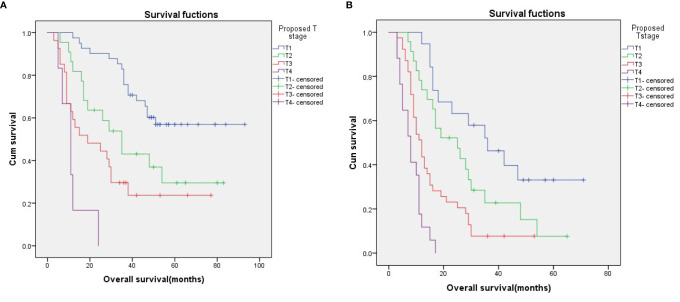
Survival curves for N0 **(A)** and N1 **(B)** patients stratified according to the proposed T stages.

To evaluate the utility of the proposed T stages in predicting survival in different N stages, we performed a stratified analysis in the N0 and N1 subgroups based on the 6^th^ edition AJCC-N staging system. The results showed that, in both the N0 and N1 subgroups, survival could be well discriminated between patients with the proposed T stages (**Figures 3A, B**; p < 0.001 for both).

### Univariate and Multivariate Analyses

In the univariate analysis, 6^th^ T stage, 6^th^ N stage, 6^th^ clinical stage, proposed T stage, modified clinical stage, chemotherapy, tumor length, and the longest and the shortest diameters on the maximum transverse plane of the tumor were prognostic predictors (**Table 1**). In the multivariate analysis, 6^th^ T stage, 6^th^ N stage, proposed T stage, modified clinical stage, and chemotherapy were independent prognostic factors (**Table 5**).

**Table 5:** Multivariate Cox Regression Analyses of the Prognostic Factors for OS in Patients with ESCC.

Variables	Hazard Ratio	95% CI	P Value
Age (>60 vs. ≤60)	0.994	0.977–1.012	0.524
Sex (Male vs. Female)	1.308	0.638–2.508	0.418
Tumor length (<7 vs.≥7cm)	1.460	0.879–2.427	0.144
Max diameter* (<3.0 vs. ≥3.0cm)	0.786	0.453–1.362	0.391
Min diameter^#^ (<1.8 vs. ≥1.8cm)	1.573	0.981–2.522	0.060
6^th^ T stage	1.353	1.022–1.791	0.035
6^th^ N stage	0.294	0.183–0.473	0.000
Proposed T stage	2.005	1.379–2.917	0.000
Modified clinical stage	0.102	0.002–0.203	0.000
Chemotherapy	0.370	0.249–0.549	0.000

^*^The longest diameter on the maximum transverse plane of the tumor.

^#^The shortest diameter on the maximum transverse plane of the tumor.

### Comparison Between the 6^th^ Edition AJCC-T and the Proposed T Staging System

According to the 6^th^ edition AJCC-T stage, there were significant differences in the 3-year OS rates among the four groups (**Figure 1A**; p=0.049). According to the proposed T stage based on tumor volume, the 3-year OS rate was also significantly different among the groups (**Figure 2A**; p < 0.001). The –2 log-likelihood ratios of the 6^th^ edition AJCC-T stage and proposed T stage were 1,068.060 and 1,047.418, respectively. The difference in the AIC value between the two T staging systems was 18.642.

## Discussion

The AJCC-TNM system is the most widely used cancer staging system worldwide and is based on the following information: primary tumor, regional lymph node, and distant metastasis (12). Nevertheless, the current 8^th^ edition AJCC-TNM staging system, which is surgical pathology-based, still has two problems for patients with non-surgical EC. First, it is difficult to accurately determine the number of metastatic lymph nodes (13); second, it is also difficult to evaluate the depth of tumor invasion accurately. Radiation oncologists prefer to use the 6^th^ edition AJCC-TNM staging system for non-surgical EC to avoid the first problem (14). For the second problem, EUS can be used to assess the thickness of the esophageal wall, which is considered to be the best procedure for determining the locoregional extension of EC (15). However, the accuracy of EUS in T staging is not satisfactory. Luo et al. (16) analyzed the staging of 112 ESCC patients and showed that the preoperative clinical staging that was assessed with EUS was not consistent with the postoperative pathological stage. In another study conducted by Barbour et al. (17), 22 of 76 (29%) EC patients with cT0-1 (preoperative EUS staging) lesions were understaged and approximately 36% of patients classified with cT2 or larger tumors were overstaged when compared with the postoperative pathological stage. Similarly, Atay et al. (18) evaluated the staging accuracy in 499 EC patients undergoing esophagectomy and found that only 14% (70/499) of patients have been staged accurately, with 50% (248/499) of patients were understaged and 36% (181/499) overstaged. Hardacker et al. (19) also found that 44% of 107 EC patients staged as cT2N0M0 with EUS were understaged. Because the accuracy of EUS classification is debatable, the AJCC-T staging system based on the depth of tumor invasion is sometimes not applicable in patients with non-surgical EC.

The effect of tumor length on the prognosis of EC has always been controversial. In the 1983 version of the AJCC-TNM staging system, the T1 stage was defined as esophageal tumor length ≤5 cm, and the T2 stage was defined as esophageal tumor length >5 cm (20). However, the tumor length was replaced by the depth of tumor invasion, which was used for T staging of EC in the subsequent 1987 version of the AJCC-TNM staging system (21). Nevertheless, neither tumor length nor tumor invasion depth performed perfectly. In 2010, the Chinese Clinical Staging Expert Group published a Clinical Staging Criteria, which considered tumor length and diameter as the basis of T staging for non-surgical patients with EC (22). However, these criteria remain in draft form and lack high-level evidence to support it. The tumor volume calculation formula that we have adopted combines the tumor length with the longest and shortest diameters on the maximum transverse plane of the tumor well. In the present study, according to the CT imaging-based tumor volume and the proposed T staging system, the 3-year OS in patients with the T1 to T4 stages was 67.9, 30.6, 21.3, and 5.3%, respectively. After the multivariate analysis, the 3-year survival rates of the proposed T stages and the modified clinical stages were also significantly different among the groups, indicating that the tumor volume was a good indicator to predict the prognosis of non-surgical ESCC patients. In addition, our study also found that the tumor length and the longest and shortest diameters on the maximum transverse plane of the tumor were all related to the prognosis, which was consistent with the results of many other studies (23, 24).

Many studies demonstrated that the tumor volume was correlated with the prognosis of patient with EC. Chen et al. (14) retrospectively analyzed the clinical data of 187 patients with EC after radiotherapy and found that the survival time in patients with high tumor volume (>39.41 cm^3^) was significantly shorter than that in patients with low tumor volume (≤39.41 cm^3^). Créhange et al. (25) calculated the tumor volumes in 148 patients with EC by assimilating and representing the esophageal tumor as two opposing truncated cones, observing that patients with tumor volume ≥100 cm^3^ had significantly worse OS than those with tumor volume <100 cm^3^. Recently, Chen et al. (26) reported that the clinical T staging based on tumor volume could accurately predict the prognosis in patients with non-surgical EC, and suggested that tumor volume should be included as a staging factor in the clinical TNM staging. In this study, we first observed the overlapping survival curves of the T stage according to the 6^th^ edition AJCC-TNM staging system. After restaging according to tumor volume, there was a significant difference among the four groups. This means that the proposed T staging method in this study surpassed the 6^th^ edition AJCC-T staging in predicting the prognosis of non-surgical ESCC patients, and the difference in AIC value between the two T staging systems was 18.642.

At present, radiation oncologists could delineate and automatically calculate the tumor volume in non-surgical EC patients who underwent radiotherapy using the treatment planning system, which was considered to be a more accurate measurement of tumor volume (27, 28). However, the correct diagnosis and exact stage classification should be completed before the determination of the treatment plan, and not after preparation for radiotherapy. Furthermore, tertiary diagnosis and treatment measures have not been fully implemented in China; most radiotherapy equipment is now allocated to developed regions, making the tumor volume measurement unfulfillable for some patients in less developed areas. In contrast, CT imaging-based measurement of tumor volume advocated in this study has better universality and applicability.

However, there are some limitations in the present study. First, it was a two-center retrospective study with a relatively small sample size. Second, for patients with non-surgical ESCC, the tumor load was always high; thus, the number of patients with T1 stage included in this study was small, which may have limited the statistical power. Third, owing to the small sample size, the cutoff values obtained in our study were not necessarily accurate, which may have also affected the applicability of the study results. Thus, our results still need to be further verified in a larger number of clinical cases.

## Conclusion

CT imaging-based tumor volume was superior to the depth of tumor invasion for T staging in predicting the prognosis of non-surgical ESCC patient treated with definitive (chemo) radiotherapy. Using tumor volume as the basis for T staging can provide a simple and accurate staging method for non-surgical ESCC patients and help clinicians make better treatment decisions. However, because of the small sample size of our study, the findings need to be confirmed by large-scale multicenter studies.

## Data Availability Statement

The raw data supporting the conclusions of this article will be made available by the authors, without undue reservation.

## Ethics Statement

The studies involving human participants were reviewed and approved by the Medical Ethics Committee of the First and the Second Affiliated Hospital of Guangxi Medical University. Written informed consent for participation was not required for this study in accordance with the national legislation and the institutional requirements.

## Author Contributions

NK, YF, and HZ wrote the initial draft and accomplished the final version. NK, LC, and YL collected the data. YF, HZ, and JL interpreted and analyzed the data. ZS and HW edited and submitted the manuscript. WL and KH developed the idea and designed the study. All authors contributed to the article and approved the submitted version.

## Funding

This work was supported by the Natural Science Foundation of Guangxi (2018JJA140869), and the Guangxi Medical and Health Appropriate Technology Development and Application Project (S2018097).

## Conflict of Interest

The authors declare that the research was conducted in the absence of any commercial or financial relationships that could be construed as a potential conflict of interest.
